# Special Issue on the 120th Anniversary of Shandong University

**DOI:** 10.1038/s41377-023-01099-1

**Published:** 2023-05-29

**Authors:** Haohai Yu, Xiangang Xu, Zhanggui Hu, Jiyang Wang, Yicheng Wu

**Affiliations:** 1 State Key Laboratory of Crystal Materials, Shandong University, Jinan, 250100 China; 2 Institute of Crystal Materials, Shandong University, Jinan, 250100 China; 3 Institute of Novel Semiconductors, Shandong University, Jinan, 250100 China; 4 Tianjin Key Laboratory of Functional Crystal Materials, Institute of Functional Crystals, Tianjin University of Technology, Tianjin, 300384 China

**Keywords:** Solid-state lasers, Nonlinear optics

This special issue is committed to celebrating the 120th Anniversary of Shandong University (SDU) (15th October, 2021), as well as capturing the most fascinating research works and reviews from all aspects of optics and photonics, including but not limited to, optoelectronic functional crystals, basic science, applied and engineering research and applications. The guest editors are five active researchers in these areas: Prof. Yicheng Wu and Prof. Zhanggui Hu from Tianjin University of Technology, Prof. Jiyang Wang, Prof. Xiangang Xu and Prof. Haohai Yu from SDU.

SDU is a key comprehensive university with a long history and continuously features creativity and dedication. It is one of the initiative universities of modern Chinese higher education and has been selected for the “Double First-class Initiative” Scheme. Its main body, Shandong Imperial College (Shandong Da Xue Tang) established in 1901, was the second national university in China. Moreover, it was the first university to be established and run in accordance with a chartered constitution. With the motto of “noble in spirit, endless in knowledge” and the mission of “nurture talent for the world and seek prosperity for the nation”, Shandong University is dedicated to the well-being of Chinese society and world development. Since the birth of the university over the years, SDU has developed at a breathtaking pace into a comprehensive research university, exhibiting great strength in research and making remarkable achievements over the years. In the fields of optoelectronic functional materials and optics, the State Key Laboratory of Crystal Materials of Shandong University is featured by the researches ranging from design and growth to device fabrication of optoelectronic functional crystals, and has supported the construction of massive national major optical projects in a long term. Meanwhile, a number of national and provincial awards have been granted, including the first prize of National Invention. To date, the State Key Laboratory of Crystal Materials of Shandong University has made great contributions to the development of national optoelectronic functional materials and the advancement of optical subjects. The discovery and development of a variety of laser and nonlinear optical crystals are famous all over the world, and the research on wide band gap semiconductor crystal materials has been on the international leading level. Furthermore, the proposed concept of optoelectronic energy materials and developed new materials has attracted extensive attention worldwide, the developed waveguides and ultra-thin functional crystals have provided important optical materials for some burgeoning research fields. This collection presents some exciting achievements in optics and optoelectronic functional crystals, contributed by SDU scholars and researchers in related fields. The innovative reports include: (1) Development of large-size optical crystals, their growth techniques and optoelectronic devices^1–3^; (2) discovery and preparation of new functional crystals and their characterization^4^; (3) innovative approaches in the laser and nonlinear optics^5^; (4) novel concepts and techniques in material and optical science^6^.

Enlightened by LSA, this special collection would stimulate more insightful perspectives in related fields and promote further progress and breakthroughs in optics, photonics, optoelectronic functional materials and beyond.
